# Supplementary figures and images for: Characterization of Polyvinyl Alcohol (PVA)/Polyacrylic Acid (PAA) Composite Film-Forming Solutions and Resulting Films as Affected by Beeswax Content
Source: Polymers (Basel). 2024 Jan 23;16(3):310. doi: 10.3390/polym16030310 (PMC10856808; doi:10.3390/polym16030310)

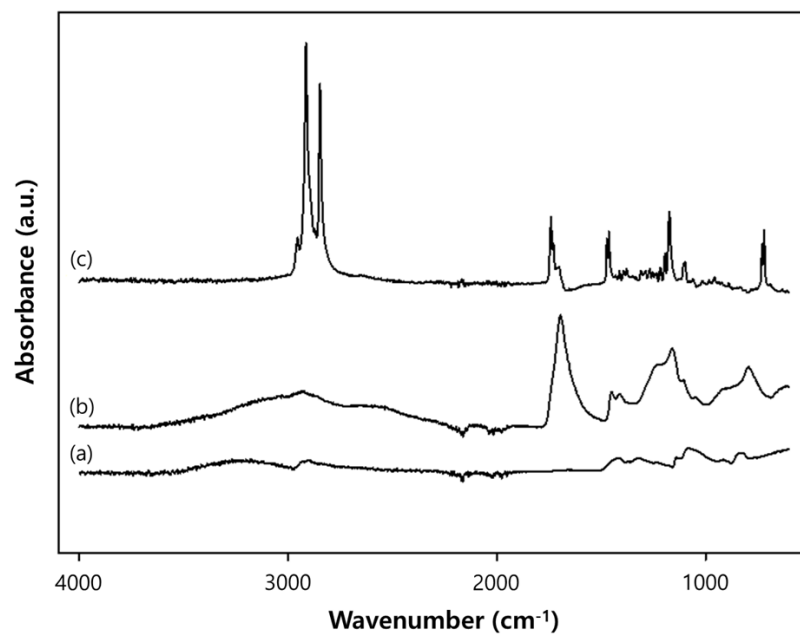

**Figure S1.** FTIR spectra of (a) PVA, (b) PAA, and (c) beeswax.

Supplement: Supplementary file 1 [file polymers-16-00310-s001.zip › polymers-2836119-supplementary.pdf]
